# Correction: Unraveling the effects of linker substitution on structural, electronic and optical properties of amorphous zeolitic imidazolate frameworks-62 (a-ZIF-62) glasses: a DFT study

**DOI:** 10.1039/d0ra90043e

**Published:** 2020-04-29

**Authors:** Mo Xiong, Xiujian Zhao, Guanchao Yin, Wai-Yim Ching, Neng Li

**Affiliations:** State Key Laboratory of Silicate Materials for Architectures, Wuhan University of Technology No. 122, Luoshi Road Wuhan 430070 P. R. China lineng@whut.edu.cn opluse@whut.edu.cn; School of Materials Science and Engineering, Wuhan University of Technology No. 122, Luoshi Road Wuhan 430070 P. R. China; Department of Physics and Astronomy, University of Missouri-Kansas City Kansas City MO 64110 USA

## Abstract

Correction for ‘Unraveling the effects of linker substitution on structural, electronic and optical properties of amorphous zeolitic imidazolate frameworks-62 (a-ZIF-62) glasses: a DFT study’ by Mo Xiong *et al.*, *RSC Adv.*, 2020, **10**, 14013–14024, DOI: 10.1039/C9RA09977H.

The authors regret that the author contributions were incorrectly given in the original article. The corrected author list and contributions are as shown above.

The Royal Society of Chemistry apologises for these errors and any consequent inconvenience to authors and readers.

## Supplementary Material

